# Necrotizing Pneumonia and Cerebral Air Embolism

**DOI:** 10.1007/s12028-023-01935-7

**Published:** 2024-02-14

**Authors:** Kai Michael Schubert, Marie-Eve Jaquier

**Affiliations:** 1 Department of Neurology and Clinical Neuroscience Center, University Hospital and University of Zurich, Frauenklinikstrasse 26, 8091 Zurich, Switzerland; 2 Department of Internal Medicine, University Hospital and University of Zurich, Zurich, Switzerland

**Keywords:** Necrotizing pneumonia, Cerebral air embolism, PVL-positive *Staphylococcus aureus*, Respiratory failure, Brain imaging, Hyperbaric oxygen therapy

**Fig. 1:**
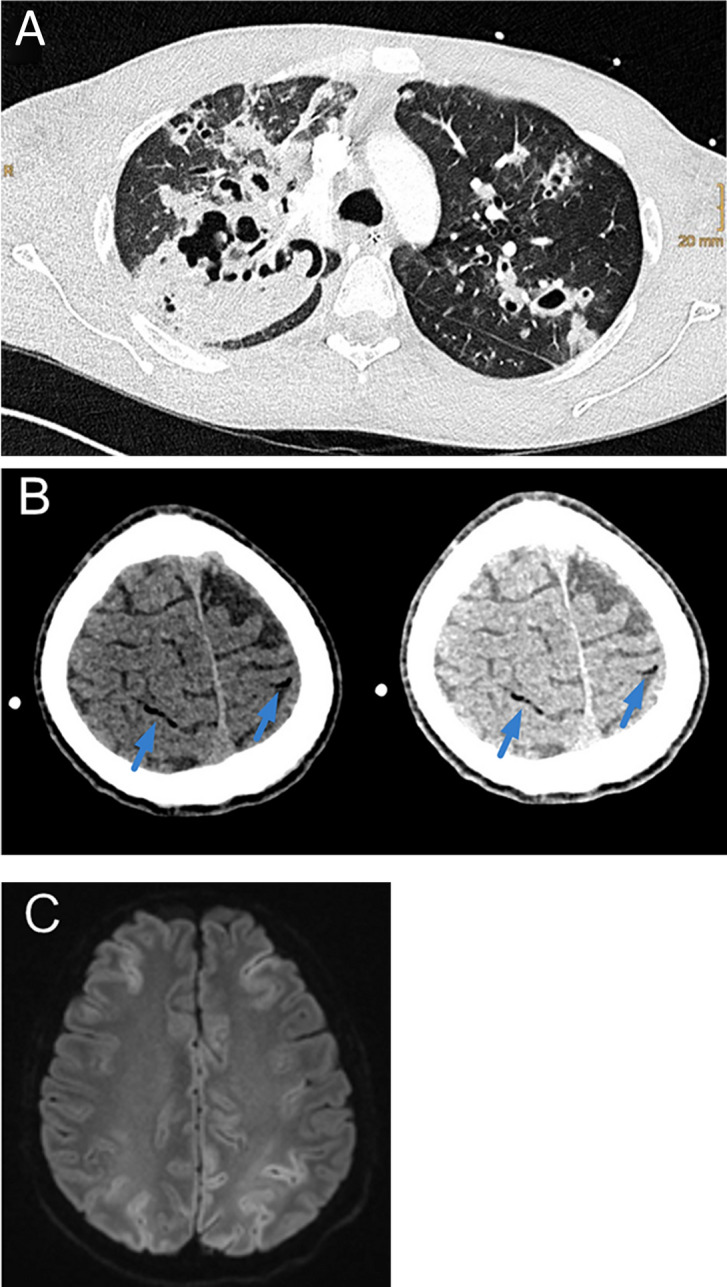
** a**, PVL-positive *Staphylococcus aureus* pneumonia stands as a prominent example of evolving virulence factors amid the era of antibiotic resistance. **b**, Cerebral air embolism often remains unnoticed when presenting new acute neurological symptoms, particularly in intensive care units. Using lung window settings (**b**, right side) compared to other standard brain windows (**b**, left side) increases the diagnostic accuracy in detecting small cerebral air embolism. **c**, Like any other embolic event, cerebral air embolism can cause tissue ischemia, as demonstrated by the diffusion restriction observed.

A 28-year-old man experienced acute respiratory failure due to PVL-positive *Staphylococcus aureus* pneumonia. Chest computed tomography (CT) showed multiple cavernous lesions indicating necrotizing pneumonia (Fig. 1a). The patient received mechanical ventilation and was treated with antibiotic therapy. After initial recovery, he suffered another respiratory failure due to aspiration during vomiting. His cardiopulmonary condition deteriorated rapidly, resulting in unconsciousness and additionally detected conjugate eye deviation. Emergency chest CT confirmed aspiration pneumonia, whereas brain CT revealed generalized bihemispheric cerebral air embolism (Fig. 1b), and a follow-up magnetic resonance imaging scan revealed cortical diffusion restriction (Fig. 1c). The air embolism likely occurred because of a right-to-left shunt from a necrotic lung lesion during his cardiopulmonary depressed state. No patent foramen ovale was found, but a positive bubble test result suggested an underlying pulmonary venous shunt. Following an extended stay in the intensive care unit, the patient was discharged to rehabilitation, with improving sensorimotor function but persistent visual deficits and neuropsychological limitations.

Cerebral air embolism warrants consideration in patients manifesting an abrupt onset of cognitive impairment, seizures, or focal neurological deficits, particularly in instances involving underlying lesions or subsequent to procedures carrying a risk of air introduction. Iatrogenic procedures, notably within the domains of vascular surgery, cardiac surgery, and neurosurgery, stand as the primary culprits for vascular air embolism, with a noticeable trend toward less invasive techniques, such as endoscopy, angiography, tissue biopsy, thoracocentesis, hemodialysis, and central/peripheral venous access. Urgent medical attention must be expeditiously administered. Placement of patients in the left lateral decubitus position and the provision of a high-flow oxygen supply constitute imperative measures. Contemplation of hyperbaric oxygen therapy to augment the elimination of air may be deemed appropriate. Prognostic implications hinge on variables such as emboli dimensions, anatomical localization, and the expeditiousness of therapeutic intervention. Thus, a prompt diagnosis and timely implementation of therapeutic measures are paramount given their potential to result in favorable clinical outcomes. Conversely, a delay in intervention may result in notable morbidity and mortality.

